# Optimal tibial component size set in unicompartmental knee arthroplasty for Chinese patients and comparison with existing implants

**DOI:** 10.1186/s13018-026-06878-1

**Published:** 2026-05-06

**Authors:** Yandong Sun, Diyang Zou, Huarui Shen, Jingchi Li, Rongshan Cheng, Pingyue Li, Tsung-Yuan Tsai

**Affiliations:** 1 School of Biomedical Engineering & Med-X Research Institute, Engineering Research Center of Digital Medicine and Clinical Translation, Shanghai Jiao Tong University, Ministry of Education, Shanghai, 200030 China; 2 MicroPort Orthopedics, Suzhou, People’s Republic of China; 3 Department of Orthopedic Surgery, Guangzhou General Hospital of Guangzhou Military Region, Guangzhou, People’s Republic of China; 4 Shanghai Key Laboratory of Orthopaedic Implants & Clinical Translation R&D Center of 3D Printing Technology, Department of Orthopaedic Surgery, Shanghai Ninth People’s Hospital, Shanghai Jiao Tong University School of Medicine, Shanghai, People’s Republic of China; 5 Department of Orthopedics, Luzhou Key Laboratory of Orthopedic Disorders, The Affiliated Traditional Chinese Medicine Hospital, Southwest Medical University, Luzhou, Sichuan Province People’s Republic of China

**Keywords:** UKA, Chinese, Anthropometry, Tibial component, Implant size mismatch

## Abstract

**Background:**

The coverage of the tibial component affects the success of unicompartmental knee arthroplasty (UKA). However, most UKA implants used in China are designed based on the knee morphology of Caucasian populations. The purposes of this study were to (1) provide accurate anatomical parameters of the medial tibial plateau in the Chinese population, (2) define an optimal set of UKA tibial component sizes based on anthropometric data, and (3) compare the tibial coverage of several commercially available UKA tibial designs with that of the suggested optimal tibial component.

**Methods:**

A standard virtual tibial resection was performed on 620 healthy knees using three-dimensional (3D) reconstructed models derived from computed tomography (CT) scans. The anteroposterior (AP) and mediolateral (ML) dimensions of the medial tibial plateau were measured. For implant sizing optimization, priority was given to the implant size that resulted in ≤ 1 mm of ML overhang; among those satisfying this criterion, the size with the smallest AP mismatch was selected. We designed sets of tibial component sizes (containing 6, 8, and 10 sizes) and optimized their configurations using a cost function defined as the weighted sum of AP and ML errors to evaluate prosthesis coverage. Commercially available UKA tibial implant designs were included to compare their mismatch errors with those of the suggested optimal tibial component.

**Results:**

In the conventional designs, the highest and lowest percentages of best-fit (within the range of -2 mm to 1 mm) in the mediolateral (ML) dimension were observed for the Zimmer Unicompartmental High Flex Knee (ZUK)/Journey (95.0%) and the Sled (41.3%), respectively. In the anteroposterior (AP) dimension, the highest and lowest percentages were found in the Oxford (49.5%) and the Journey (15.3%). In the three optimal designs, the percentages of best-fit in the ML dimension were 83.1%, 86.0%, and 88.1% for the 6-, 8-, and 10-size sets, respectively. In the AP dimension, the corresponding percentages were 55.5%, 72.6%, and 77.1%.

**Conclusion:**

Most commercially available UKA tibial component size sets cannot fully accommodate the anatomical variation of the tibia in the Chinese population. Ethnic-specific designs similar to the optimal designs proposed in this study are needed.

## Introduction

Unicompartmental knee arthroplasty (UKA) is an effective and widely used treatment for end-stage unicompartmental osteoarthritis (OA) [15, 17, 31]. The success of UKA procedures has been shown to depend significantly on the fit of the tibial component [3, 10]. However, most UKA systems offer only 5 to 8 tray sizes with fixed increments in the anteroposterior (AP) and mediolateral (ML) dimensions [3, 19]. Surgeons must select the most suitable tibial prosthesis from this limited range to avoid excessive overhang or underhang. An undersized tibial implant increases the risk of subsidence and loosening [4, 13, 33], while an oversized implant may cause soft tissue irritation and pain [4, 13].

Currently, the majority of UKA implants used in China are based on the knee morphology of Caucasian populations. However, studies have demonstrated significant differences in proximal tibial anatomy between Caucasian and Asian populations [18, 24, 26, 35]. Yue et al. reported that Chinese individuals have smaller proximal tibiae with distinct aspect ratios compared to Caucasians [35]. Similarly, Kim et al. found notable differences in AP length and ML width between these groups [18]. These morphological disparities suggest that existing tibial implant designs may not be suitable for Chinese patients, thereby increasing the risk of component mismatch.

Several studies have examined tibial implant mismatch in Asian populations using commercially available UKA designs [16, 19, 22]. Lu et al. analyzed 1,000 subjects using magnetic resonance (MR) imaging and found that 71.3% of resected surfaces did not fit current UKA tibial components [22]. Koh et al. reported similar mismatches in Korean patients, with ML overhang occurring in three out of five conventional UKA designs [19]. While these studies highlight the inadequacy of existing implants for Asian populations, they do not propose anatomical refinements or improved prosthesis designs.

Some researchers have attempted to enhance tibial coverage by modifying component shapes, such as teardrop, D-shaped, or patient-specific designs [12, 20]. However, achieving an optimal fit requires not only shape adjustments but also careful consideration of size distribution. For surgeons, a broader range of sizes reduces the gaps between increments, thereby improving the likelihood of an ideal fit. Conversely, manufacturers and hospitals face logistical and financial challenges in producing and stocking excessive component variations. Furthermore, most conventional UKA tibial tray designs currently on the market offer a limited range of sizes with fixed, often irregular, increments that do not align with the anatomical distribution of Asian populations, leading to poor fit for certain sizes [16, 19, 22].

Although meticulous preoperative planning, including the use of appropriately scaled radiographs, and skillful intraoperative adjustments can help accommodate minor implant-resection mismatches and optimize component positioning, these surgical maneuvers cannot fully overcome inherent anatomical discrepancies caused by a lack of appropriately sized implants. To the best of our knowledge, only one study has attempted to design the size distribution of the tibial component in total knee arthroplasty (TKA) using an optimized method to achieve better fit for more patients [5]. However, the “best-fit circle” method with a 5 mm radius used for TKA is unsuitable for UKA due to differing design constraints [4, 5, 13]. Moreover, minor mismatches (1–2 mm) can often be mitigated through strategic surgical planning, such as adjusting component placement relative to the intercondylar eminence. Preoperative planning with appropriately scaled radiographs is critical for anticipating and addressing potential mismatches, emphasizing that optimal outcomes depend not only on implant availability but also on meticulous surgical technique.

This study aims to (1) design an optimized medial tibial component size set tailored to Chinese patients and (2) compare coverage errors between traditional commercial implants and the proposed optimized size sets, balancing clinical needs with manufacturing feasibility.

## Materials and methods

### Participants

This study was approved by the Institutional Review Board of Guangzhou General Hospital of Guangzhou Military Region, and the approval number is Academy Ethics [2019] No. 10. All data were collected from the hospital’s image database. CT scans from patients with neuromuscular (i.e., non-skeletal) injuries were retrospectively analyzed after obtaining institutional approval. A total of 620 patients were included to investigate the anatomical coverage of the tibial prosthesis after simulated osteotomy (Table 1). The mean age was 41.3 ± 17.5 years (range: 17–93 years), mean height was 166.2 ± 9.5 cm (range: 142–190 cm), mean weight was 67.0 ± 11.6 kg (range: 38–105 kg), and mean BMI was 23.7 ± 5.1 kg/m² (range: 16.3–35.4 kg/m²).

**Table 1:** Anthropometry of subjects

Parameter	Total (*n* = 620) Mean ± SD	Male (*n* = 338) Mean ± SD	Female (*n* = 282) Mean ± SD
Side (n)	Left (*n* = 310) Right (*n* = 310)	Left (*n* = 169) Right (*n* = 169)	Left (*n* = 141) Right (*n* = 141)
Age (years)	41.3 ± 17.5	34.3 ± 14.6	49.6 ± 17.2
Height (cm)	166.2 ± 9.5	173.1 ± 6.0	157.9 ± 5.6
Weight (kg)	67.0 ± 11.6	72.6 ± 10.1	60.4 ± 9.6
BMI (kg/m^2^)	24.2 ± 3.5	24.2 ± 3.3	24.2 ± 3.7

SD, Standard deviation; BMI, Body mass index

## Reconstruction and resected tibial measurement

**Fig. 1:**
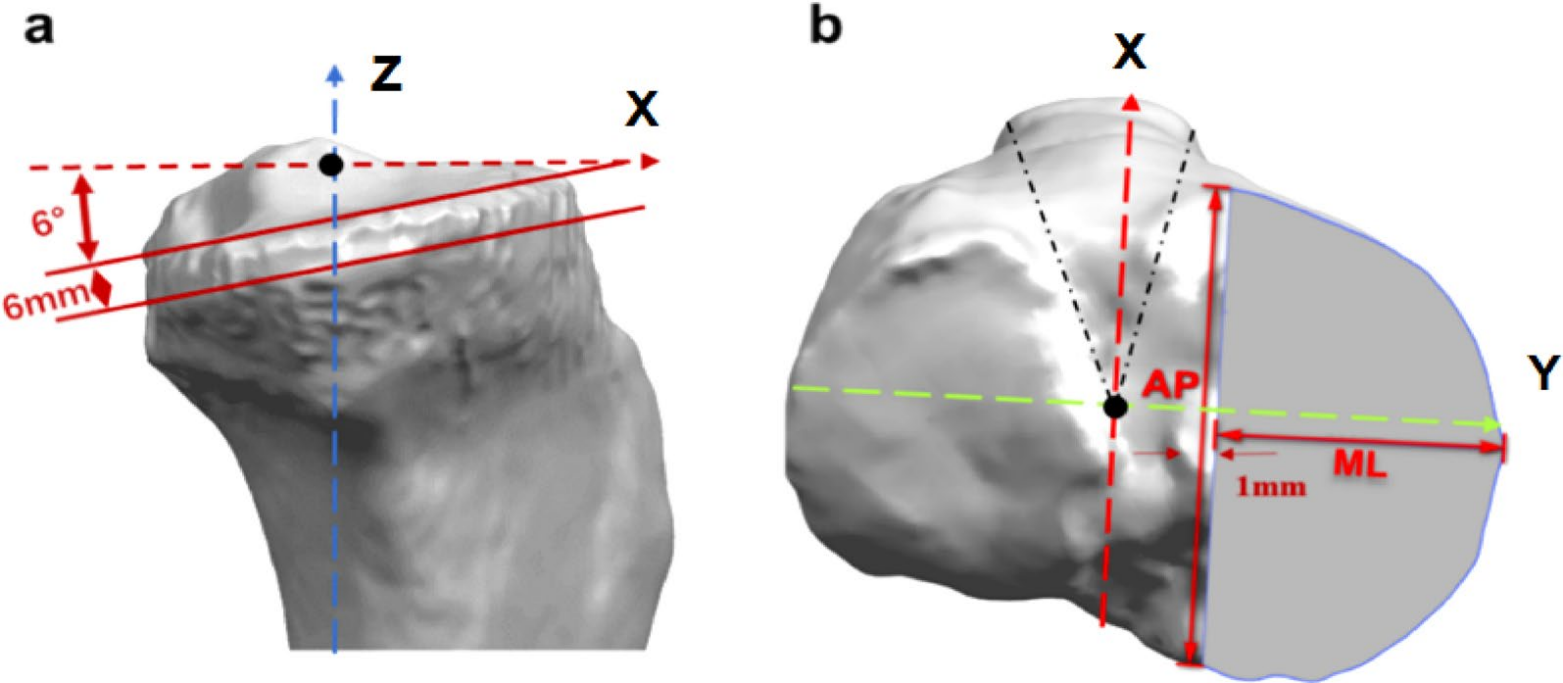
Virtual medial tibial condylar cuts and measurement methods using 3D reconstructed model. **a** Thickness of the tibial cut is 6 mm and the posterior slope is **b** an anteroposterior cut was made parallel to X direction and 1 mm apart to highest point of medial intercondylar tubercles

All subjects underwent CT scanning (Discovery CT750 HD, GE Medical Systems, USA) from the hip to the ankle joints, with high-resolution image reconstruction (120 kVp; axial image resolution: 512 × 512 pixels; slice thickness: 0.6 mm; voxel size: 0.86 × 0.86 × 0.63 mm³). Three-dimensional (3D) models of the proximal tibia were reconstructed from CT images using commercial modeling software (Amira 6.7.0, Thermo Fisher Scientific, Hillsboro, OR, USA). An anatomical coordinate system was established to guide the proximal tibial resection (Fig. 1). The origin of the tibial coordinate system was defined as the center of the tibial plateau. The X-axis was defined as the line connecting the point at the medial one-third of the tibial tubercle and the origin. The Y-axis was defined as the line perpendicular to the X-axis and passing through the origin. The Z-axis was defined as the line connecting the origin and the center of the ankle joint, perpendicular to both the X- and Y-axes.

To ensure accuracy and consistency, a standard virtual tibial resection was performed by an experienced examiner using Siemens NX software (Version 11.0, Siemens, Germany) within the established anatomical coordinate system. Although different implant systems may have varying requirements for resection thickness and posterior slope angle, this study aimed to compare anatomical coverage under consistent resection parameters. As described by Carpenter et al., commonly used values of 6 mm resection thickness and a 6° posterior slope were adopted [3]. An anteroposterior (AP) cut was then made parallel to the AP-axis (X-axis) and positioned 1 mm medial to the apex of the medial intercondylar eminence. The AP and mediolateral (ML) dimensions of the resected tibial surfaces were subsequently measured [25] (Fig. 1). A two-way mixed-effects model for intraclass correlation coefficient (ICC) with absolute agreement yielded an ICC (3,1) of 0.99, indicating excellent reliability.

## Optimal tibial component size set design

**Fig. 2:**
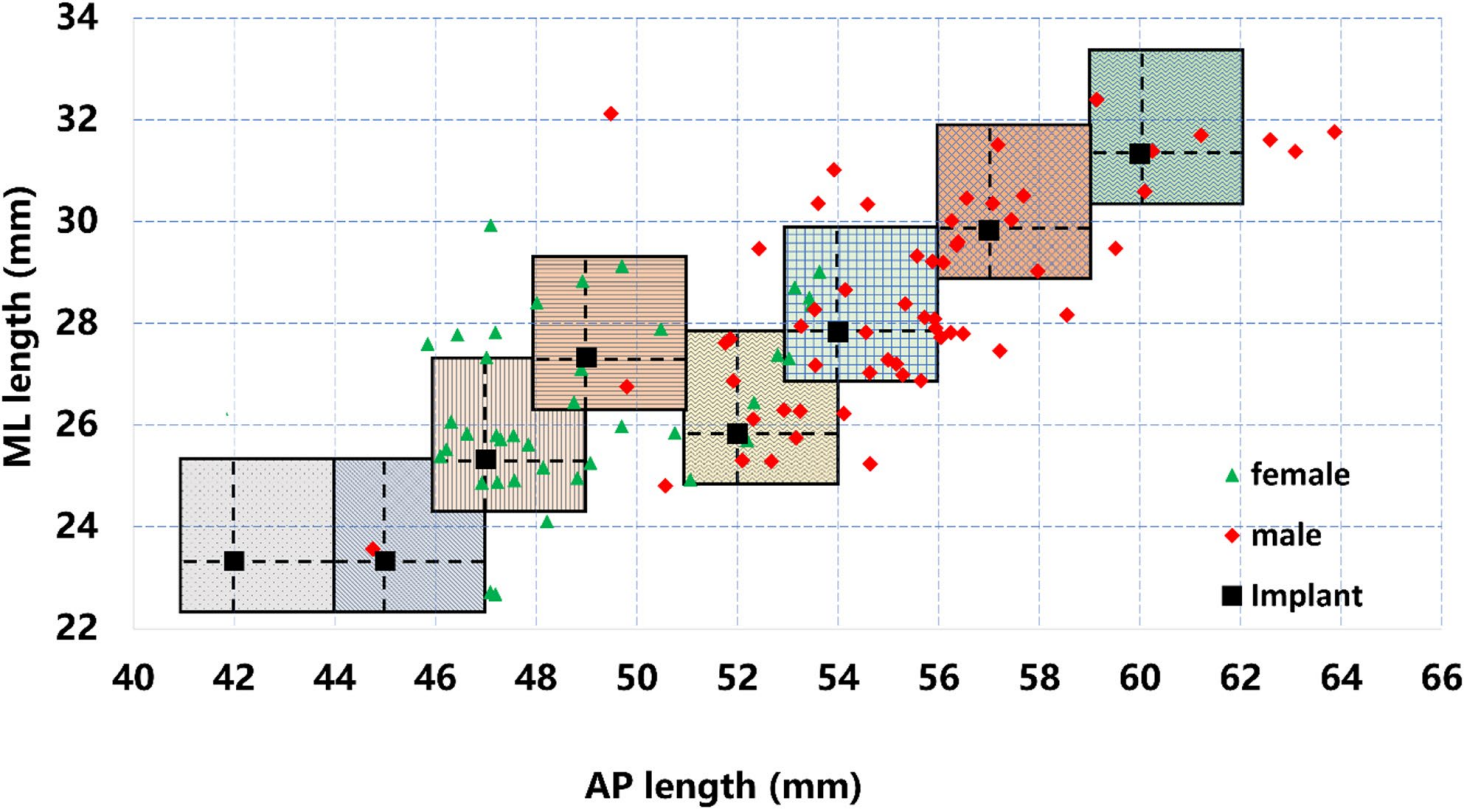
The tibial implant chosen rule for each subject. One of the design sizes set of tibial implants was illustrated in black square. The bounding box around each size of implant represented its best-fit range in value of no more than 2 mm and no less than 1 mm in AP and ML dimension. The green and red point represented the size of medial tibial plateau for female and male subjects. The star point represents one of the subjects and $\:{e}_{i}^{AP}$and $\:{e}_{i}^{ML}$ represented the tibial coverage error of selected subject in AP and ML dimension

To design the optimal tibial component size, it was first necessary to establish selection criteria for each subject. In this study, the principle of prioritizing mediolateral (ML) fit was applied. This involved selecting the prosthesis with the closest ML dimension, allowing a maximum overhang of 1 mm, while also choosing the prosthesis that best matched the anteroposterior (AP) dimension for each subject. Three sets of tibial implant sizes were designed, containing 6, 8, and 10 sizes, respectively. The distribution of prosthesis sizes within each set adhered to specific constraints. The incremental gap between adjacent sizes was limited to − 2 mm, − 1.5 mm, 0 mm, 1.5 mm, or 2 mm in the ML dimension for all size sets. In the AP dimension, for the 6-size set, increments were restricted to 0 mm, 2 mm, 3 mm, or 4 mm; for the 8- and 10-size sets, increments were limited to 0 mm, 2 mm, or 3 mm. The initial AP size of the tibial implant in each set was constrained to a range of 41 to 50 mm, with sizes incrementing by 1 mm. The initial aspect ratio (AP length / ML length) was limited to 1.5, 1.6, 1.7, 1.8, 1.9, or 2.0. The corresponding initial ML size was then calculated based on the initial AP size and aspect ratio (Fig. 2). For example, with an initial AP size of 42 mm, an aspect ratio of 1.8, and fixed AP and ML increments of 2 mm and 1.5 mm, a complete size set could be generated based on these parameters. A cost function, defined as the sum of the weighted errors in each subject’s AP and ML dimensions, was used to optimize the size distribution of each set. A brute-force optimization method was then employed to determine the optimal size sets for the 6-, 8-, and 10-size configurations.

Six conventional UKA tibial designs were included for comparison to evaluate mismatch errors relative to the current design: (1) ZUK (Zimmer, Warsaw, IN, USA), (2) MCK (Stryker, Kalamazoo, MI, USA), (3) Journey (Smith & Nephew Inc., Memphis, TN, USA), (4) Sled (Waldemar Link, Hamburg, Germany), (5) Preservation (DePuy-Johnson and Johnson, Warsaw, IN, USA), and (6) Oxford (Zimmer Biomet, Warsaw, IN, USA).

All measured data were imported into a custom program developed in MATLAB (Version 2020a, MathWorks, Natick, MA, USA) to determine the best fit. The optimal solution was obtained by minimizing a weighted sum of AP and ML errors (Eq. 1), where αi and (1 − αi) represent the weighting factors (Eq. 2).

Formula 1: $$\:\mathrm{E}=\mathrm{a}\mathrm{r}\mathrm{g}\mathrm{m}\mathrm{i}\mathrm{n}\{\sum\:_{i=1}^{n}\left({\left(1-{\alpha\:}_{i})e\right.}_{i}^{AP}+{{\alpha\:}_{i}e}_{i}^{ML}\right)$$

Formula 2: Weight: $$\:\frac{1-{\alpha\:}_{i}}{{\alpha\:}_{i}}=\frac{M{L}_{i}}{A{P}_{i}}=\frac{1}{{\lambda\:}_{i}}$$

## Commercial implant size data and comparison methodology

To ensure a standardized and equitable comparison with commercially available UKA systems, the nominal AP and ML dimensions (defined as the maximum outer boundary lengths of the component baseplate) for all available sizes of the six investigated systems (ZUK, MCK, Journey, Sled, Preservation, Oxford) were obtained from their latest official surgical technique guides or product catalogs.

The core objective of this comparison was to evaluate the two-dimensional size coverage, i.e., the ability of a prosthesis size series to match the measured AP/ML dimensions of a resected bone surface, rather than to assess three-dimensional shape, fixation features, or specific surgical protocols. Although these commercial systems differ in tray geometry (e.g., symmetrical vs. anatomical, presence of a posterior cutout), fixation mechanism, and surgical philosophy, they share a fundamental requirement: the component must adequately cover the resected bony surface to minimize the risks associated with overhang or underhang [4, 13]. Therefore, for the purpose of this comparative coverage analysis, we adopted the premise that, under ideal fitting conditions, the prosthesis’s nominal AP/ML footprint should approximate the prepared bone surface. This approach allows for a focused assessment of how well the size distribution of each system matches the anatomical variation in our population.

For each virtual resection model in our dataset (*n* = 620), its measured AP and ML dimensions were programmatically matched against the complete size matrix of each commercial system. The “best-fit” prosthesis for a given knee was selected according to the same “ML-priority” rule defined in our optimization algorithm: first, identify all sizes with an ML overhang ≤ 1 mm, then from this subset, select the size with the smallest absolute AP error. The resulting AP and ML mismatch values (implant size minus bone size) were recorded for each knee and each implant system. This process quantified the population-scale coverage performance of each commercial size set.

Special Note on the Oxford Implant: The Oxford Partial Knee system employs a unique sizing scheme where multiple AP sizes are offered for a single ML width. In our analysis, we treated each unique AP/ML combination listed in the manufacturer’s sizing chart as a distinct size option during the “best-fit” selection process. This approach fully accounts for its design specificity when evaluating coverage rates.

### Statistical analysis

Intraclass correlation coefficients (ICCs) were calculated to quantify interobserver and intraobserver agreement of the measurements. Twenty randomly selected knees were chosen for repeated measurements by the main observer after a one-month interval. A second observer also repeated the measurements for the same set of knees. Knee dimensions were presented as means and standard deviations. An independent-sample t-test was used to compare differences between male and female subjects. All statistical analyses were performed using MATLAB (MATLAB 2020a, MathWorks, Natick, MA, USA). The significance level was set at *p* < 0.05.

Due to the high statistical power inherent in our large dataset (*n* = 620), formal hypothesis testing was omitted, as even clinically irrelevant differences would yield statistical significance. Consequently, we prioritized descriptive statistics to focus on the magnitude of the geometric mismatch rather than statistical probability.

## Results

### Medial tibial morphology of the Chinese population

The morphological analysis of the medial tibial plateau in the Chinese population demonstrated excellent measurement reliability (ICC: 0.99/0.98 for intraobserver/ interobserver AP length; 0.99/0.98 for ML width) and revealed significant gender differences (*p* < 0.05). Males showed larger average dimensions (AP: 56.9 ± 3.4 mm; ML: 29.6 ± 2.2 mm) compared to females (AP: 53.7 ± 4.8 mm; ML: 28.2 ± 2.6 mm). Males also exhibited a slightly smaller AP/ML aspect ratio (1.93 ± 0.12 vs. 1.88 ± 0.14), a clinically critical parameter, as it represents the intrinsic proportionality of the tibia that cannot be surgically adjusted, unlike ML width, which can be modified during implantation (Table 2).

**Table 2:** Asian morphology reported in previous studies

Authors	Population	Dimension	Male (mm)	Female (mm)	Total (mm)
Our study	Chinese (338 male and 282 female)	AP*	56.9 ± 3.4	49.8 ± 2.9	53.7 ± 4.8
		ML*	29.6 ± 2.2	26.6 ± 2.2	28.2 ± 2.6
		AP/ML*	1.93 ± 0.12	1.88 ± 0.14	1.91 ± 0.13
Dai [7]	Indian (50 male and 47 female)	AP	51.7 ± 2.4	45.6 ± 2.6	–
		ML*	–	–	–
		AP/ML*	–	–	–
Dai [7]	Japanese (44 male and 55 female)	AP	52.4 ± 2.3	46.3 ± 2.1	–
		ML*	–	–	–
		AP/ML*	–	–	–
Surendran et al. [30]	Korean (50 male and 50 female)	AP	49.8 ± 3.3	44.3 ± 2.6	47.1 ± 4.1
		ML	26.1 ± 1.9	23.5 ± 2.2	24.8 ± 2.5
		AP/ML	1.91	1.88	1.9 ± 0.1
Koh et al. [19]	Korean (149 male and 814 female)	AP*	55.9 ± 4.0	49.5 ± 3.3	50.5 ± 4.0
		ML*	29.9 ± 3.2	27.7 ± 3.3	28.1 ± 3.4
		AP/ML*	1.9 ± 0.2	1.8 ± 0.2	1.8 ± 0.2
Cheng et al. [6]	Chinese (94 male and 78 female)	AP	52.1 ± 2.7	47.1 ± 2.8	49.8 ± 3.7
		ML	29.3 ± 2.0	27.0 ± 1.9	28.2 ± 2.2
		AP/ML	1.78	1.74	1.8 ± 0.1
Lu et al. [22]	Chinese (500 male and 500 female)	AP*	51.8 ± 2.9	47.0 ± 2.9	49.5 ± 3.8
		ML*	29.1 ± 2.1	24.3 ± 2.0	26.7 ± 3.2
		AP/ML*	1.79	1.92	/

*Significant difference between male and female (*p* < 0.05)

## Tibial plateau and implant size distribution in the Chinese population

**Fig. 3:**
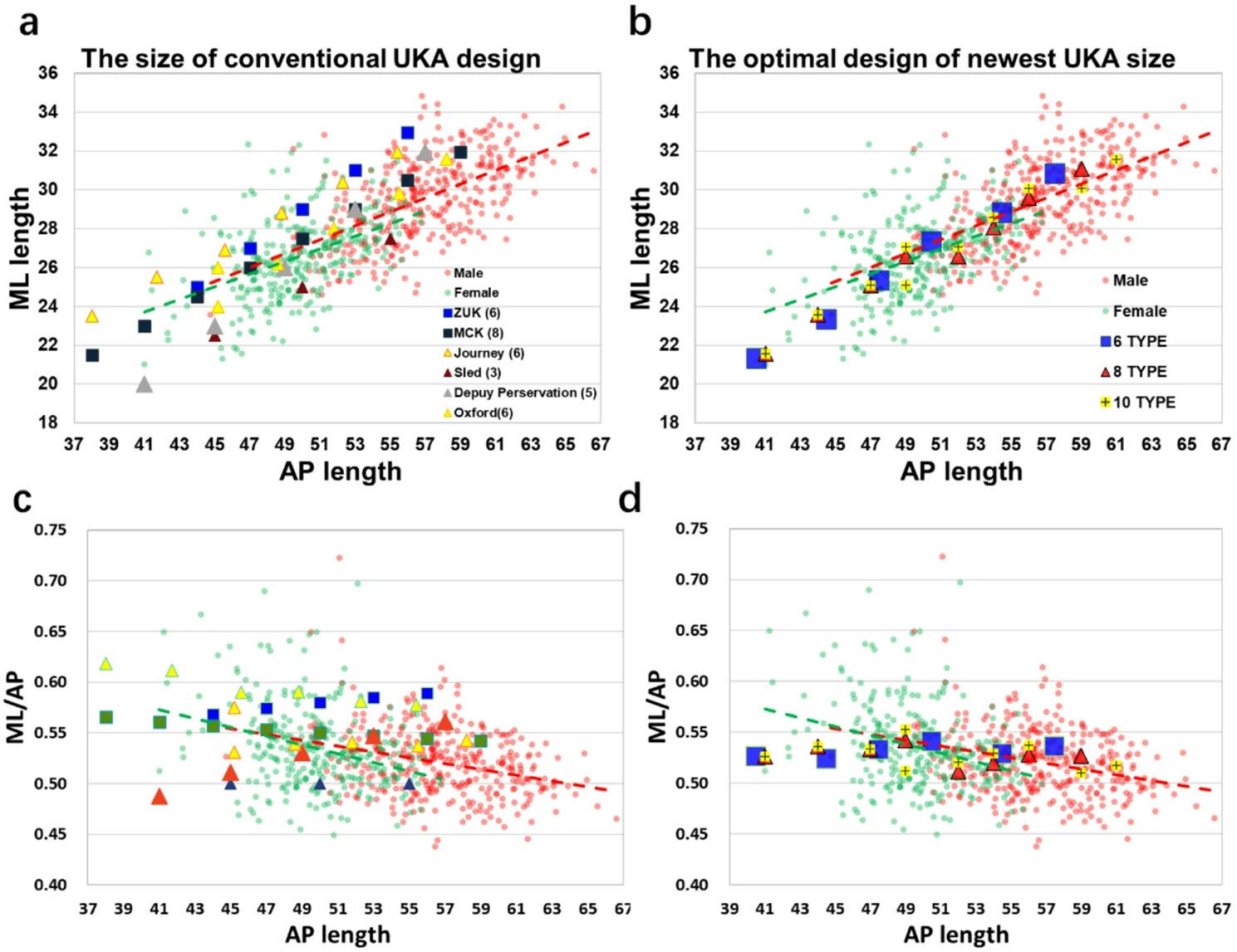
Anteroposterior (AP) and mediolateral (ML) direction size distribution of the six conventional UKA designs (**a**, **c**) and the three optimal UKA designs (**b**, **d**). The red and green points represent the medial plateau size of male and female respectively. The six conventional designs (ZUK, MCK, Journey, Sled, DePuy Preservation and Oxford) were represented by different markers, The number after the name indicates the quantity of that product model. The red and green dashed lines represent the linear regression curves for the size of the medial tibial plateau in males and females respectively

Evaluation of the six conventional UKA designs revealed substantial variation in their conformity to Chinese tibial morphology (Fig. 3), with three distinct patterns emerging: (1) Ratio-compatible designs, including Preservation and MCK, demonstrated the closest approximation to the native anatomy of the Chinese population, whereas the optimized designs (6-, 8-, and 10-size sets) exhibited a distribution of sizes that evenly matched the central tendency of the population; (2) Among the ratio-deviant designs, the Sled maintained a consistently high aspect ratio exceeding native values, while the Journey and Preservation exhibited aspect ratios that decreased with increasing size, and the ZUK and Journey designs clustered in the upper region of the scatterplot. Notably, although Oxford’s unique provision of multiple AP sizes for each ML width improved coverage, it failed to accommodate the smaller tibiae that are common in this population - a limitation that was successfully addressed by the optimized designs while maintaining aspect ratio fidelity (Fig. 3b and d).

### Quantification of size error

Size mismatch analysis revealed critical limitations in conventional UKA designs, with severe AP underhang (< − 3 mm) observed in 73.5% of ZUK and 76.8% of Journey cases. Best-fit rates ranged from just 15.3% (Journey) to 49.5% (Oxford), although Oxford exhibited the lowest no-fit rate (3.2%) despite substantial size gaps. In the ML dimension, best-fit rates varied widely (from 41.3% to 95.0%); however, these mismatches are less clinically concerning due to the potential for intraoperative adjustment. In contrast, the optimized designs demonstrated progressive improvement: AP best-fit increased from 55.5% (6-size set) to 77.1% (10-size set), while ML best-fit remained above 83% across all variants, and no-fit scenarios were completely eliminated. These findings illustrate how an expanded range of prosthesis options can better accommodate anatomical variation, particularly in the surgically uncorrectable AP dimension.

## Discussion

The most important finding of this study is that most commercially available UKA tibial component size sets do not fully accommodate the anatomical variation of the tibia in the Chinese population. Ethnic-specific designs similar to the optimal designs proposed in this study are needed. Our comparative analysis, which evaluated coverage based on the nominal footprint dimensions of commercial implants, revealed that even the best-performing existing designs showed significant mismatch rates. Based on the morphology of the medial tibial plateau in the Chinese population, the use of an exhaustive optimization method to determine the distribution of prosthesis sizes can effectively minimize mismatch errors in UKA.

In our study, the AP and ML dimensions of the medial tibial plateau were 53.7 ± 4.8 mm and 28.2 ± 2.6 mm, respectively—larger than those reported for Korean (50.5 ± 4.0 mm for AP and 28.1 ± 3.4 mm for ML) and Chinese populations (49.5 ± 3.8 mm for AP and 26.7 ± 3.2 mm for ML) in previous studies [19, 22]. This discrepancy may be attributed to differences in sample size, patient height, and measurement methodologies. Furthermore, numerous morphological studies have demonstrated significant variations in tibial plateau morphology and dimensions between Asian and Western populations [14, 23, 35]. In a 2017 study, Ma et al. identified ethnic differences in the slope angle, AP length, ML width, and aspect ratio of the proximal tibia. Their findings indicated that Asian populations generally exhibit smaller tibial dimensions and higher aspect ratios compared to Caucasian populations [23]. Additionally, several studies have reported gender-specific differences in tibial morphology, with males exhibiting larger tibial dimensions and higher aspect ratios than females [2, 6, 36]. These findings are consistent with the results observed in our study. Currently, the majority of UKA implants are designed based on morphological measurements from Caucasian populations. Ro et al. reported a higher complication rate when using the same UKA prostheses in Chinese patients compared to Caucasian populations [27]. In light of our findings, we propose that population-specific and gender-specific implant designs may improve outcomes in unicompartmental knee arthroplasty.

The mismatch between the tibial component and the resected surface is a critical factor contributing to complications such as postoperative pain and subsidence at the implant-bone interface in UKA [32–34]. An ideal tibial component should be perfectly flush with the edges of the resected tibial surface. However, because standard implants may not provide the optimal shape and size for every patient, surgeons must strike a balance between component overhang and underhang. In this study, the optimal range for prosthesis-bone matching was defined as overhang of less than 1 mm and underhang of less than 2 mm. Chau et al. reported that only 3% of patients received a perfectly sized tibial component, and their study indicated that tibial overhang exceeding 3 mm leads to a significant increase in pain and a decline in clinical function of the knee joint. Therefore, surgeons should avoid tibial component overhang of 3 mm or more during prosthesis selection [4]. Another in vitro study demonstrated no significant difference in medial collateral ligament (MCL) loading between specimens with no overhang and those with 2 mm of overhang, concluding that tibial component overhang should be limited to less than 2 mm [13]. Simpson et al. found that strain in knees with 2 mm of tibial implant overhang was higher than that in the neutral model [29]. Ahmed et al. further categorized component overhang, concluding that overhang of less than 1 mm represents a close approximation of physiological conditions [1]. Therefore, an overhang of less than 1 mm was considered the optimal threshold for prosthesis-bone matching in this study.

Previous studies have suggested that tibial component overhang may lead to adverse outcomes, including knee pain, reduced functional mobility, and stiffness, which are generally unacceptable to clinicians [10]. As a result, selecting a smaller prosthesis size and adjusting its rotational positioning to achieve improved anteroposterior component-to-bone coverage has become a common strategy in UKA procedures [28]. Schroeder et al. reported that the thickness of the tibial cortical bone is typically around 1.5 mm [28]. Fitz et al. argued that a UKA component should be no more than 2 mm smaller than the native knee dimension; otherwise, insufficient cortical bone support may lead to prosthetic subsidence [11]. Similarly, Lee et al. found that an oversized component is more likely to cause imbalances in knee joint pressure and ligament tension, whereas an appropriately sized or slightly undersized component may help prevent valgus deformity resulting from loading imbalances [21]. Therefore, limiting tibial component undersizing to within 2 mm is considered an optimal approach in UKA.

**Fig. 4:**
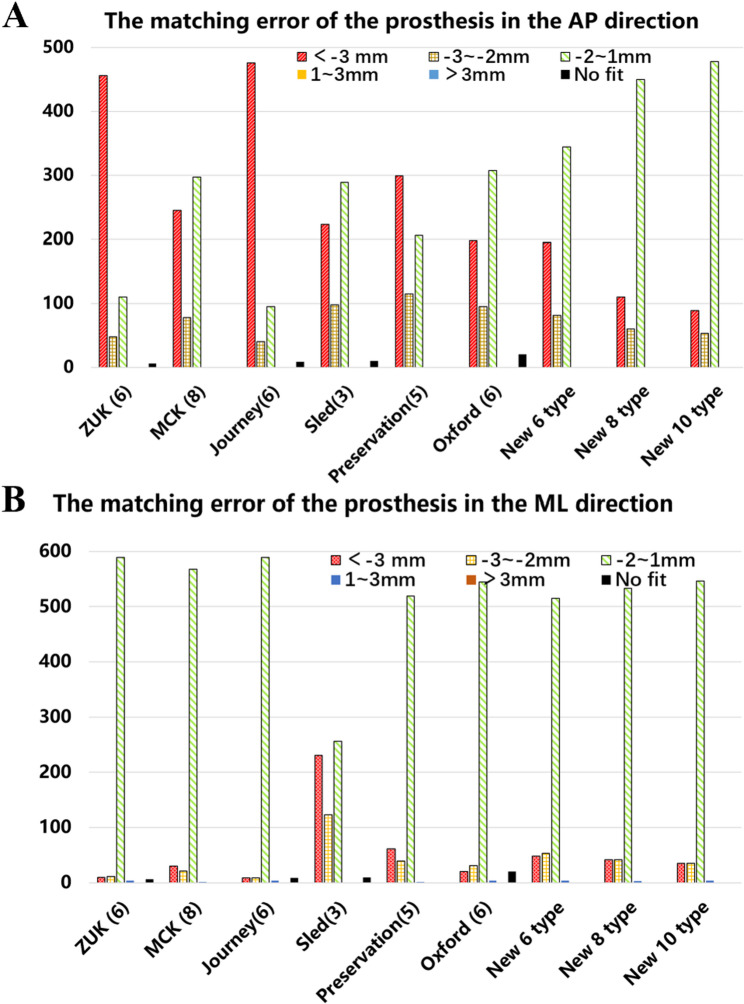
Comparison of the number of subjects of coverage error in different values in (**a**) ML width and (**b**) AP length

The size distribution of the six conventional UKA designs (Fig. 3a) and their corresponding matching errors (Fig. 4) clearly demonstrated that, among all evaluated designs, the Oxford prosthesis was the most suitable for the Chinese population. It achieved high compatibility when considering both ML and AP dimensions simultaneously (Fig. 4). The Oxford design was one of the few that featured multiple AP and ML increments and was the only design offering different AP sizes under the same ML size. It demonstrated good matching in the ML dimension (87.9%) and acceptable alignment in the AP dimension (49.5%). However, a study by Lu et al. reported that 71.3% of patients had resection surfaces that did not match the Oxford UKA component, particularly for larger prosthesis sizes [22]. The study suggested that the ML/AP ratio of the Oxford prosthesis increases with AP size, whereas the actual ML/AP ratio among Chinese individuals tends to decrease as AP increases, leading to size mismatch [22]. In the present study, larger-sized Oxford prostheses were positioned above the size trend line (Fig. 3c), which is consistent with the findings of Lu et al. Koh et al. compared the fit of various commercial prostheses in the Korean population and found that the ZUK prosthesis aligned more closely with tibial morphological distribution than other designs, while the Preservation prosthesis showed the poorest match [19]. In contrast, the Sled design was distributed in the lower region of the plot (Fig. 3a) and featured only three sizes with large, fixed AP and ML increments. As a result, its overall size errors in both ML and AP dimensions were substantial (Fig. 4; Table 3). Regarding the Preservation and MCK designs, it appeared that for a given increase in the AP dimension of the resected medial tibial surface, the corresponding increase in ML dimension was smaller in the Chinese population (Fig. 3a). Current evidence indicates that the size distribution of commercially available UKA prostheses does not fully accommodate the anatomical characteristics of the Chinese tibia, suggesting significant potential for improvement in prosthesis size design.

**Table 3:** The matching errors in the ML andAP directions of the current commercial UKA prostheses and the prosthesis designed in this study

Error (mm)	ZUK (6)	MCK (8)	Journey (6)	Sled (3)	Perservation (5)	Oxford (6)	New 6 type	New 8 type	New 10 type
*The percentage of coverage error in medial/lateral direction*									
<− 3	1.6%	4.8%	1.5%	37.3%	9.8%	3.2%	7.7%	6.8%	5.6%
− 3~− 2	1.8%	3.4%	1.5%	19.8%	6.3%	5.0%	8.5%	6.8%	5.6%
− 2~ 1	95.0%	91.6%	95.0%	41.3%	83.7%	87.9%	83.1%	86.0%	88.1%
1~3	0.6%	0.2%	0.6%	0.0%	0.2%	0.6%	0.6%	0.5%	0.6%
>3	0.0%	0.0%	0.0%	0.0%	0.0%	0.0%	0.0%	0.0%	0.0%
No fit	1.0%	0.0%	1.5%	1.6%	0.0%	3.2%	0.0%	0.0%	0.0%
*The percentage of coverage error in anterior/posterior direction*									
<− 3	73.5%	39.5%	76.8%	36.0%	48.2%	31.9%	31.5%	17.7%	14.4%
− 3~− 2	7.7%	12.6%	6.5%	15.8%	18.5%	15.3%	13.1%	9.7%	8.5%
− 2~1	17.7%	47.9%	15.3%	46.6%	33.2%	49.5%	55.5%	72.6%	77.1%
1 ~ 3	0.0%	0.0%	0.0%	0.0%	0.0%	0.0%	0.0%	0.0%	0.0%
>3	0.0%	0.0%	0.0%	0.0%	0.0%	0.0%	0.0%	0.0%	0.0%
No fit	1.0%	0.0%	1.5%	1.6%	0.0%	3.2%	0.0%	0.0%	0.0%

AP, Anteroposterior; ML, Mediolateral

Various studies have investigated the extent of mismatch when currently available commercial UKA tibial designs are applied in Asian populations. These studies have emphasized the need to consider tibial plateau characteristics and gender differences [9, 18, 24, 35]. Fitzpatrick et al. employed statistical techniques to design the optimal theoretical shape of a unicompartmental tibial prosthesis and demonstrated that asymmetric prostheses provide maximal cortical bone coverage while achieving an optimal balance between ease of manufacturing and coverage [12]. Cheng et al. previously used an optimization method with a 5 mm diameter circle as the coverage standard for TKA prosthesis size distribution design, although their study did not consider constraints between prosthesis sizes [5]. Based on measured medial tibial morphology, we have designed a new UKA tibial component size set that achieves higher AP dimension matching while prioritizing good ML dimension matching. This approach effectively improves anatomical coverage in the Chinese population. The data show that the optimized size set has a significantly better probability of achieving a fit within the − 2 mm to 1 mm range than all traditional designs. Dai’s study emphasized that offering a more diverse range of femoral prosthesis sizes and shapes for different ethnic groups and populations in TKA could significantly improve surgical outcomes and patient satisfaction [8]. The present study similarly highlights that providing more size options can enhance component-to-bone matching and reduce the proportion of prosthetic overhang or underhang greater than 2 mm in Chinese patients, which is consistent with the findings of Yue et al. in TKA [37]. We also found that as the number of prosthesis sizes increases, the matching percentage of the optimal 8- and 10-size sets obtained through optimization methods also increases. When expanding from a 6-size to an 8-size set, the improvement in size set coverage is particularly pronounced. However, expanding the size range also entails drawbacks and costs, such as increased production complexity, higher inventory demands, and potential logistical challenges.

This study has several limitations. First, we assessed the anthropometry of the resected medial tibial plateau using a single resection thickness of 6 mm and a posterior slope of 6° for the entire population, as this represents the most commonly employed resection method. Further research could explore different resection levels and orientations. Second, this study focused on the AP and ML size designs of the tibial component, which are critical factors influencing the risk of soft tissue impingement or inflammation. However, more comprehensive and sophisticated parameters could be examined in future studies. Third, although the closest implant size was determined based on the ML dimension, given that ML oversizing has been shown to contribute to loss of flexion and pain, it is important to note that ML fit can be surgically adjusted by a few millimeters through lateral positioning, potentially allowing the use of a wider component. Therefore, the priority of the AP dimension in implant selection warrants further investigation.

## Conclusion

This study provides accurate anatomical parameters of the medial tibial plateau and quantitatively evaluates the two-dimensional coverage errors of six commercially available UKA designs, including the ZUK, MCK, Journey, Sled, Preservation, and Oxford. The significance of this study lies in its finding that conventional UKA designs are not suitable for a large proportion of the Chinese population, highlighting the need for ethnic-specific designs. Furthermore, we have developed a feasible method for optimizing the size distribution of UKA tibial components, specifically aimed at reducing coverage errors such as underhang and overhang observed when traditional prostheses are used in Chinese patients. This methodological approach may also be applicable to other ethnic populations to minimize similar clinical risks, provided that the same anatomical measurement and size distribution principles are followed.

## Data Availability

The data used in the current study are available from the corresponding author upon reasonable request.

## Abbreviations

UKA: Unicompartmental knee arthroplasty
3D: Three-dimensional
CT: Computed tomography
AP: Anteroposterior
ML: Mediolateral
ZUK: Zimmer unicompartmental high flex knee
OA: Osteoarthritis
MR: Magnetic resonance
TKA: Total knee arthroplasty
MCK: Multicompartmental knee
ICC: Intraclass correlation coefficients
MCL: Medial collateral ligament
